# The Unexplored Role of Intra-articular Adipose Tissue in the Homeostasis and Pathology of Articular Joints

**DOI:** 10.3389/fvets.2018.00035

**Published:** 2018-03-05

**Authors:** Luminita Labusca, Florin Zugun-Eloae

**Affiliations:** ^1^National Institute of Research and Development for Technical Physics, Iasi, Romania; ^2^Orthopedics and Traumatology, Emergency County Hospital Saint Spiridon, Iasi, Romania; ^3^Immunology and Genetics, Grigore T. Popa University of Medicine and Pharmacy, Ias‚i, Romania; ^4^Regional Institute of Oncology Iasi - IRO, Ias‚i, Romania

**Keywords:** intra-articular fat pad, Hoffa fat pad, white adipose tissue, cellular therapies, osteoarthritis

## Abstract

Intra-articular adipose tissue deposits known as articular fat pads (AFPs) are described to exist within synovial joints. Their assumed role in normal joint biomechanics is increasingly objectivized by means of advanced methods of functional imaging. AFPs possess structural similarity with body subcutaneous white adipose tissue (WAT), however, seems to be regulated by independent metabolic loops. AFP dimension are conserved during extreme WAT states: obesity, metabolic syndrome, lipodystrophy, and cachexia. Hoffa fat pad (HFP) in the knee is increasingly recognized as a major player in pathological joint states such as anterior knee pain and osteoarthritis. HFP contains numerous population of mesenchymal and endothelial progenitors; however, the possible role of mature adipocytes in the maintenance of stem cell niche is unknown. We propose that AFP is an active component of the joint organ with multifunctional roles in the maintenance of joint homeostasis. Endowed with a rich network of sensitive nervous fibbers, AFPs may act as a proprioceptive organ. Adipokines and growth factors released by AFP-resident mature adipocytes could participate in the maintenance of progenitor stem cell niche as well as in local immune regulation. AFP metabolism may be locally controlled, correlated with but independent of WAT homeostasis. The identification of AFP role in normal joint turnover and its possible implication in pathological states could deliver diagnostic and therapeutic targets. Drug and/or cell therapies that restore AFP structure and function could become the next step in the design of disease modifying therapies for disabling joint conditions such as osteoarthritis and inflammatory arthritis.

## Introduction

White adipose tissue (WAT) is increasingly recognized as a multifunctional, metabolically active organ (1). The evolutionary conserved attribute of storing excess energy as lipid deposits is coupled with WAT role in controlling metabolic balance as a body-wide distributed endocrine organ (2). By accumulating nutrients deposits, mature WAT resident cells—the adipocytes—fulfill a basic life function necessary to provide energy during periods of high caloric demands. However, far from being mere inert warehouses, the adipocytes not only store triacylglycerol but secrete as well regulatory bioactive molecules such as adipokines (leptin, adiponectin, resistin, adipsin, and visfatin), cytokines (IL-6 and TNF-α), and acylation-stimulating protein. Such bioactive molecules have local, peripheral, and central effects in controlling nutrient intake, energy storage, and expenditure (3). The multifactorial role WAT posses in coordinating and executing a diversity of organismal functions is reflected by its heterogeneous cellularity. WAT is composed not only of adipocytes but harbors a variety of blood cells, immune resident and endothelial elements, pericytes, and adipose precursor cells as well as mesenchymal progenitor cells. Various enzymatic or mechanical methods can be used to separate the so called stromal vascular fraction (SVF)—a mixture of mononuclear elements among which adipose derived mesenchymal stromal cells (ADSCs).

Mature adipocytes together with WAT resident macrophages are active players in local and systemic immune response by releasing pro-inflammatory cytokines and adipokines that orchestrate local and central pathways of the innate immune system (4). The endothelial cells, pericytes together with adipose precursor cells are responsive of the angiogenetic and expansive capabilities WAT displays (5). In the last decades, WAT has been recognized as a reservoir of ADSCs and sought as convenient, easy accessible, source for cellular therapies (6). Complex physiological roles of WAT in lipid and glucose metabolism, coagulation, appetite regulation, angiogenesis, body weight control, and reproduction have been well described and documented [for review, see Ref. (2)].

White adipose tissue is widely distributed in almost the entire body subcutaneous region, in organs and hollow viscera of the abdominal cavity, in mediastinum as well as in several muscle groups functioning as a thermal insulator and shock absorber. Its role in mechanical protection has been related to the lax extracellular matrix (ECM) structure and to the important capability to recover from mechanical deformation (7).

White adipose tissue structure and function varies with body distribution. Subcutaneous fat presents distinct cellular and sec-retory profile compared to visceral fat (8). Regions where fat deposits might serve a mere mechanical role—soles, palms, and periarticular deposits—seem to have a particular genetic profile as they are conserved in some forms of congenital generalized lipodystrophy (9).

However, despite WAT heterogeneity in structure and function, its responsiveness appears to be closely correlated by similar, if not the same, neuroendocrine and biochemical pathways. Shared biochemical profile during pathological states obesity and metabolic syndrome at one pole and lipodystrophy and cachexia at the opposite pole draws the picture of a body-wide organ functioning as an organismal network with possible site-specific adaptive particularities.

## Articular Fat Pad (AFP) is a Multifunctional Tissue within the Normal Joint Organ

An interesting and potentially important WAT location has been, surprisingly, largely neglected. AFPs have been mainly mentioned in the context of pathological joint states (such as knee pain and osteoarthritis—OA) (10) or as a source of progenitor and stem cells (11). Their potential roles in maintaining homeostasis in normal joints remains unexplored. It remains obscure if AFP function and metabolic profile is correlated with systemic WAT normal and pathological states or is regulated by potential separate mechanism connected or not to the biomechanical function within the joint.

This paper will introduce the hypothesis of AFP as an internal homeostatic joint regulator, in possible relation but distinctive from body WAT function and balance. We propose that AFP is a specialized tissue of the joint organ contributing to its homeostasis by releasing bioactive molecules implicated in cell and ECM growth, turnover, and repair. AFP biomechanical role and its function in joint homeostasis are intertwined and might be locally regulated and systemically coordinated. AFP might act by converting information about joint biomechanics (alignment, axis, and dynamics) into biochemical cues that contributes to regulating the homeostasis of all articular tissues.

## The Anatomy—AFP, Ubiquitous Presence in Synovial and Non-Synovial Joints

Commonly, intra and periarticular fat deposits are included within the category of joint adjacent supportive structures together with menisci and ligaments. Such structures, described to be heterogeneously present in some joints (hip, knee) are thought to contribute to joint stability and to function as a shock absorber (12).

**Figure 1:**
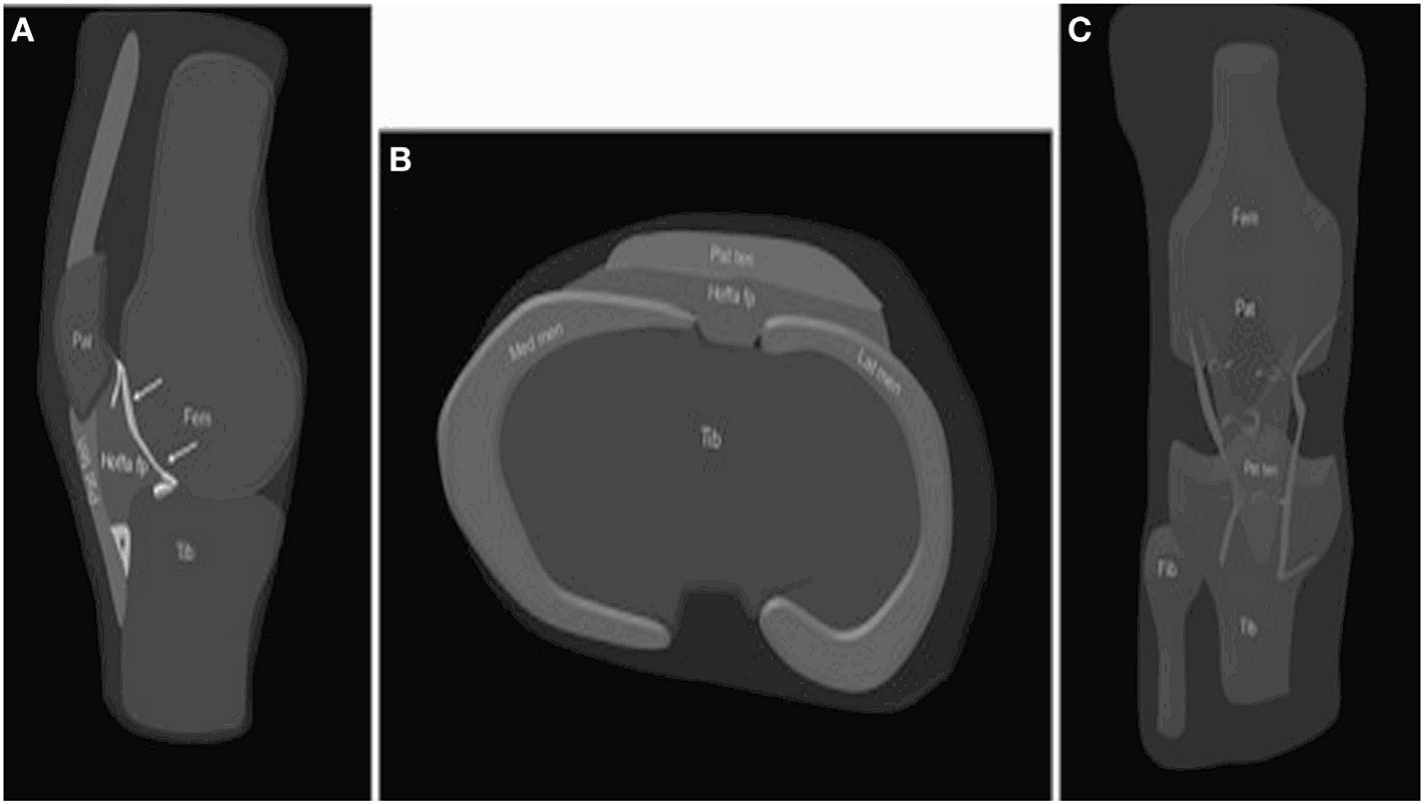
Anatomy of the HFP. The HFP (*Hoffa fp*) is limited anteriorly by the patellar tendon (PT) (*Pat ten*) and the joint capsule, superiorly by the inferior pole of the patella (*Pat*) **(A)**, inferiorly by the proximal tibia (*Tib*) and the deep infrapatellar bursa (*asterisk*), and posteriorly by the synovium (*arrows*) and femur (*Fem*). It is attached directly to the anterior horns of the menisci (*Med men, Lat men*) **(B)**. Normal vascular supply consists of two vertical arteries, posterior and parallel to the lateral edges of the PT **(C)**. Courtesy of Draghi et al. from Insights into Imaging 2016 (15).

**Figure 2:**
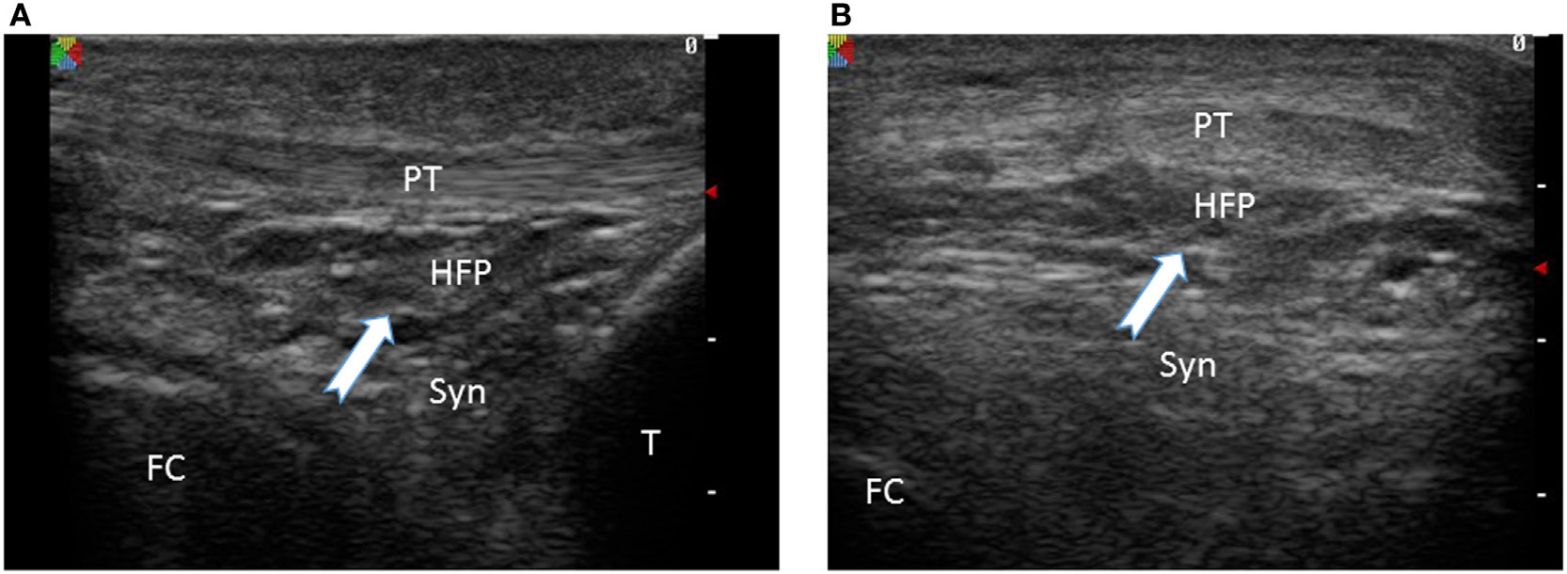
Ultrasound image of infrapatellar fat pad of a normal knee joint White arrow indicates the fat tissue with distinct ultrasound features compared to the underlying patellar tendon (PT) and synovial tissue (Syn). HFP, Hoffa fat pad; FC, femoral condyle; T, tibia. **(A)** Sagittal view, **(B)** coronal view.

Possibly the largest AFP in humans, the infrapatellar fat pad of the knee joint, known as Hoffa’s fat pad (HFP) (13) is one of the three fat pads of the knee joint interposed between the capsular layer and the synovium, described as intra-articular (intracapsular) but extra synovial structures. HFP is delimited superiorly by the inferior pole of the patella, inferiorly by the tibial bone, intermeniscal ligament, meniscal horns, and infrapatellar bursa, anteriorly by the patellar tendon while posteriorly is bounded by the femoral condyles and the intercondylar notch. It occupies the entire anterior part of the joint in all knee positions (14). Macroscopically, HFP is composed of a fibrous scaffold filled with fat lobules containing as well a number of septae such as the infrapatellar plica (IPP) (also known as the ligamentum mucosum) (15). A clinically relevant classification of HFP variants based on the presence or the absence of IPP has been proposed (Class I, HFP constrained, IPP present; and, Class II, FP unconstrained, no IPP), suggesting class I HFP might function as an intra-articular ligament involved in joint stability (16). Similarly with WAT, HFP cellularity consist of mature adipocytes but include as well fibroblasts, macrophages, and leukocytes within a lax network of conjunctive tissue and a rich network of blood vessels. As a particularity, the presence of peptidergic C-fibers, nerve fibers suggests HFP role as a sensory organ. HFP has anatomical, histological, and imagistic characteristic that distinguish it from underlying synovial tissue (Figures 1 and 2) (17). HFP is sought to act as a deformable space filler that adapts to the changing articular contours during joint movement and to facilitate synovial joint distribution. HFP is seen as a contributor to anterior knee pain probably due to an impingement mechanism after joint trauma (18) and as a source of inflammation and disease progression in knee OA (10). By far the most known, HFP is not, however, the sole AFP.

In the hip joint, a relatively recently identified structure placed at the anterior head–neck junction of the upper femur has AFP characteristics and is sought to be a source of femoroacetabular impingement (19). Fat pads opposite the olecranon, coronoid, and radial fosse as well as fibroadipose meniscoids in the nonarticular waists of the trochlear notch and into the posterolateral aspect of the radiohumeral joint were described in 28 normal adult cadaveric elbows and proposed to be starting points in arthrofibrosis (20).

Articular fat pads were described to be present superior and inferior to lumbar facet joints from which fat-filled synovial folds project between the articular surfaces the superior being intracapsular between the ligamentum flavum and the lamina while the inferior remains extracapsular lying on the back of the lamina below and communicates with the joint through a hole in the inferior capsule. Lumbar facets AFPs can be identified by computer tomography scans. Lumbar facets AFPs enlargement is sought to be associated with degenerative changes and capsular laxity of the facet joints (21).

Intermetacarpal fat pads have been described to be adipose structures located between the heads of the second, third, fourth, and fifth metacarpal bones, filling the spaces between the palmar fascia and its deep expansions. They are proposed to act as protection from shear forces during gripping, to protect neurovascular finger bundles, and to contribute to neurological symptoms when inflamed or injured (22).

The development of modern imagistic such as ultrasound and arthroscopy made possible not only the identification of previously unknown AFPs location but enabled as well direct visualization, dynamic assessment, and quantification of their biomechanical role within the joint.

## The Physiology—AFP—Simple Cushion or Multifunctional Regulator?

It is noteworthy to mention that historically, “synovial fat pads” were regarded as the sources of synovial fluid (SF). AFPs were denominated harversian glands and thought to produce substances that “oil” the joint surface. Currently, the name is kept only in regard with the acetabular floor AFP (known as harversian fat). The attributed physiological role in joint lubrication was only later replaced by an assumed mechanical filler-cushioning function.

The existence of physiological particularities of the intra-articular deposits distinguishing this tissue from widespread subcutaneous fat was suspected by anatomists as late as the middle of the twentieth century. Despite the structural similarity to body-wide WAT, AFPs persist even during advanced states of malnutrition, when all other deposits are depleted. This observation raised the hypothesis that such tissue might possess yet unexplored particular ultrastructural and secretory features of potential importance for joint function (23).

However, the study of fat pat deposits seem to have fallen into oblivion until late 1990s when sports physicians and physiotherapists become interested in its involvement in producing (especially knee) joint pain. Reports related almost, if not completely to HFP, are stressing out the involvement of its rich nervous network and abundant substance P fibers in producing anterior knee pain in various pathological states (18, 19, 24). Noteworthy, until not too long ago, AFP biomechanical role was simple assumption. Very few biomechanical studies were conducted to address directly the role of adipose structures within or adjacent to joints. Indirect evidence points toward inflammatory reactivity of HFP during patellar mal tracking correlated with trochlear morphology and patella alignment (25). Moreover, static imaging or cadaveric studies make it difficult to assess the dynamics of articular structures. With the development of high resolution ultrasound probes, fat pad kinesiology within the knee joint and the mechanism of impingement could be explored (26). Kager’s fat pad located in Kager’s triangle between the Achilles tendon, the superior cortex of the calcaneus, and flexor hallucis longus muscle and tendon has been reported to perform important biomechanical functions that are crucial for the maintenance of ankle posterior tendons as well as ankle joint. Using high-resolution dynamic ultrasound and electromyogram, Kager fat was shown to lubricate the subtendinous region, to reduce the pressure change within the Achilles tendon enthesis, and to remove debris from within the retro calcaneal bursa (27). Due to advances in functional imaging, the assumed role of AFP and its participation to the joint biomechanics is increasingly documented. Its importance in the normal functioning of several joints is increasingly acknowledged. Little is known, however, about the possible participation of AFP in joint mechanical balance. Could it be possible that similar to other musculoskeletal structures, AFP possess a proprioceptive role contributing to dynamic alignment of the structures around the joint? Further studies about the presence of such proprioceptive receptors within AFP could elucidate this question.

## Possible Links between Adipose Tissue Metabolism and Joint Function

Recent years have broadened understanding about the role of local and systemic hormonal balance in maintaining joint health (28). With increasing understanding about the WAT function as an endocrine organ, a growing list of adipokines with pleiotropic local and systemic actions are investigated in relation to musculoskeletal tissue biology (29). Adipokines have been implicated in a bidirectional bone—energy metabolism interplay, in the central regulation of bone mass as well as in the fatty bone marrow metabolism (30). Leptin levels are correlated with WAT mass, functioning as a food intake and energy consumption regulator. Leptin has been demonstrated to play crucial roles in influencing prenatal development and postnatal growth as well as in modulating systemic immune response. In humans, leptin deficiency or resistance is implicated in the pathogeny of obesity, metabolic syndrome, diabetes, and infertility. Leptin is expressed not only by adipocytes but as well by osteoblasts and chondrocytes and contribute to regulating chondrocyte differentiation and matrix maturation during enchondral bone formation (31). Human and murine chondrocytes express leptin and adiponectin both *in vivo* and *in vitro* (32) in normal conditions. Cultured and native normal human chondrocytes express leptin receptor b, shown to modulate expression of Frizzled-1 and Frizzled-7 in a possible cross talk with canonical Wnt signaling pathway that could be implicated in cartilage homeostasis (33). Not surprisingly, HFP express leptin however to date, the majority of existent data result from investigating pathological joint states (see below). Mainly studied in relation to joint degenerative processes. Leptin is known to stimulate inflammatory cytokine production (such as interleukine β Ilβ), to induce expression of matrix degradative peptides (such as matrix metalloproteinases—MMPs) and to activate nitric oxide synthase. Leptin facilitates the activation of macrophages, neutrophils, dendritic cells, and natural killer cells contributing to establishing an inflammatory milieu within OA joints (34). Little is known about the mechanisms of leptin production by AFP and its levels in normal joints. SF leptin levels have been shown to fluctuate in correlation to body mass index (BMI) as well as with knee OA stages (35) however not correlated with plasma leptin levels (36) suggesting an independent regulatory mechanism within the joint. Moreover, existent basic science studies on normal osteoblast and chondrocyte development and metabolic homeostasis points toward an independent intra-articular regulatory mechanism of leptin levels that could have AFP as central point. Another well-studied adipokine, adiponectin, possess divergent roles in metabolism and musculoskeletal biology, being implicated in bone loss and inflammation-mediated matrix degradation (29). Human normal chondrocytes express functional adiponectin receptors that under specific stimulation were shown to express pro-inflammatory cytokines and nitric oxide synthase type II (37). Adiponectin SF levels were found, however, to be lower in female subjects with OA compared to plasma levels while higher levels could be recorded in rheumatoid arthritis (RA) compared to OA joints. Adiponectin could have anti-inflammatory role in RA by counteracting the pro-inflammatory role of tumor necrosis factor alpha (TNF-α), mechanism not reproduced in OA patients. While the multifaceted role of adiponectin in RA progression still needs to be clarified, to date there is no evidence that intra-articular and serum levels are correlated or that the protein can cross the capsular barrier to enter the joint. Intra-articular adiponectin was found to be released by synovial tissue, HFP, and even by osteophytes (38). Here again, there is a scarcity of data collected from normal joints. If adiponectin intervene in joint homeostasis or its release is solely an adaptive mechanism triggered by the presence of inflammatory mediators, needs to be further elucidated. Other adipokines such as resistin and visfatin were shown to be present in SF or plasma of RA or/and OA patients mainly in correlation with increased pro-inflammatory cytokines levels TNFα or interleukin 6. Their role as possible mediators of destructive joint inflammation is under investigation (39).

Adipokines are involved in normal joint development and possibly participate to adult bone and cartilage homeostasis. As resulting from epidemiological studies, intra-articular levels in diseased joints do not correlate with plasma serum levels. Due to obvious ethical limitations, very few information exist to profile the adipokine levels in normal joints in subjects with various BMI values. It is yet unknown if intra-articular adipokines in normal and pathological joints are produced by local elements or originate in WAT with different location (such as subcutaneous fat). In the absence of relevant information, AFP contribution to the homeostasis of the normal joints remains unknown. Conversely, AFP involvement in pathological joint conditions is increasingly stressed out and suggests a local regulatory mechanism in close dialog with the well-known inflammatory milieu that characterizes such diseases. The interplay between AFP biomechanical and secretory function may serve as a turning point between joint dynamics, axis, and alignment and the control of local metabolism, under the influence but possible distinct from body nutritional status. Deciphering the cross talk between AFP as a bio mechanic sensory organ that responds by modulating joint organ turnover and controlling local inflammation could contribute to increased understanding of joint functioning.

## AFP—A Possible Role in Maintaining Articular Stem Cell Niche

Tissue niches are known to control site-specific stem cell function, governing their transition from quiescence to proliferation and maturation. Mature adipocytes were shown to contribute to the maintenance of stem cell niche in various locations and to generate niches for other cell types. Mature adipocytes within bone marrow were shown to inhibit hematopoetic stem cell engraftment (40). Conversely, adipocytes were proved to upregulate the branching and development of mammary gland epithelium (41). Adipocyte precursors may promote muscle differentiation since interaction between muscle cells and adipogenic PDGFR alpha(+) mesenchymal progenitors has a considerable positive impact on muscle turnover (42). Mature adipocytes were shown to be necessary and sufficient for the activation of skin epithelial stem cells (43).

HFP was shown to represent a rich source of ADSCS or perivascular stem cells with superior chondrogenic potential compared to the subcutaneous fat pad (44). HFP-derived stem cells from diseased knee states maintain their chondrogenic potential *in vitro* suggesting a conserved cartilage progenitor pool might exist within the tissue (45).

The mechanism by which HFP contributes to controlling the decision of intra-articular stem cells of various origin to entering cell cycle and differentiation remains to be established. Local release of growth factors (GFs) that trigger stem cell activation or an indirect immune-mediated contribution could be involved. Indeed IL-10-producing type 1 regulatory T cells (Tregs) were shown to modulate the activity of mice MSCs in a mice model of RA (46). Further *in vivo* studies are needed to confirm the interplay between local Tregs and mesenchymal progenitor in normal joint states and disease as well as the possible influence of intra-articular mature adipocytes in maintaining this balance.

## Hoffa Fat Pad—An Active Player in Knee Pain and Osteoarthritis

By far the most investigated AFP, HFP begins to develop in humans starting with the 11th gestational week from the mesenchymal tissue below the patella, between the cruciate and the patellar ligaments (38). Its structure is very similar with subcutaneous WAT, however, does not fluctuate quantitatively with caloric intake, persisting even in severe cases of malnutrition and do not increase with BMI in obese subjects. Noteworthy, impaired functionality of HFP mature adipose cells with decreased adipose-related markers PPARγ together with increased fibrosis and macrophage infiltration could be demonstrated in obese compared to lean patients during late-stage OA of the knee (47). HFP has been implicated in a direct manner in occurrence of persistent anterior knee pain during local trauma or impingement syndrome. Its involvement in the development and progression of joint degenerative diseases is proposed to be multifactorial. Sensory tissue innervation and the contribution to increasing immune cell amount and activity within the joint are doubled by the role of pro-inflammatory adipokines are proposed as mechanisms of knee joint degeneration.

HFP is a very sensitive structure due to the presence of peptidergic C-fibers, nerve fibers staining positive for substance P (48) that are implicated in the development of knee pain after repetitive trauma in athletes and in painful knee OA (49). Moreover, substance P-induced Hoffa pad vasodilatation and immune cell extravasation could be the mechanisms of fat pad edema documented in patients with Lyme arthritis (50). The disturbed balance between substance P fibbers and sympathetic nerve fibers releasing anti-inflammatory cytokines and endogenous opioids was implicated in RA knee pain or in painful total knee arthroplasty. Besides their role in neuropathic sensitization, P fibers could have a direct pro-inflammatory effect that ignite and maintain OA development (51).

As it is the case with synovial tissue, HFP is the stage of immune cells invasion during OA and RA that triggers production of pro-inflammatory and pro fibrotic cytokines from local activated macrophages. After the initiation of joint degradation, cartilage breakdown molecules could activate monocytes and trigger innate immunity mechanisms (52). HFP-resident macrophages could produce various GFs, cytokines, and enzymes having as effect osteophyte formation, cartilage breakdown by MMPs activity, joint effusion by vasodilation, and perturbed subchondral bone metabolism.

The role of WAT produced adipokines in the initiation and aggravation of inflammatory processes at systemic level as well as within the joint is well established. Leptin, adiponectin, and resistin were reportedly found in SF of OA and RA patients at concentrations that differ from blood levels. Such pro-inflammatory mediators could be produced by HFP by an independent locally regulatory mechanism that is not correlated with body fat and nutritional status.

Pertaining to the largest joint in the body, HFP contribution to knee pain and pathological conditions is increasingly recognized. If, however, there is a connection between biomechanical joint misbalance and HFP function in contributing and sustaining joint inflammatory milieu, has not yet been established. Dynamic biomechanical studies could elucidate if potential HFP function as a proprioceptive sensor correlates with its secretory role and contributes to both joint organ maintenance. Its perturbed functional states could generate targets for complex joint re-balancing.

## WAT and Its Extremes—Implication for AFP Functioning

Obesity and metabolic syndrome are currently recognized to generate systemic and peripheral pro- inflammatory status. Moreover, OA has been proposed to be a metabolic disease associated with the chronic low-grade inflammation that defines obesity and metabolic syndrome and with the impaired cartilage homeostasis in the context of lipid and glucose abnormalities (53). Far less is known about systemic and local WAT metabolism and pathways that are involved in involuntary adipose tissue loss—lipodystrophy and cachexia.

Lipodystrophy is defined as the acquired or genetically induced partial or complete loss of metabolically active WAT. Age or disease-associated cachexia are wasting syndromes associating severe fat and muscle loss (54). As a common denominator, all pathological systemic involuntary WAT loss cannot be reversed by nutrition. Many of the metabolic impairments associated with obesity and metabolic syndrome are shared between the two extremes. Insulin resistance, glucose intolerance, and systemic inflammation are common findings in both excess and waste of systemic WAT (55). The particularities of joint metabolism during extreme adipose tissue loss are less understood. Disabling decrease in joint and body mobility is generally attributed to severe muscle loss and disturbed energy metabolism. During RA, progressive stage of the disease is associated with progressive fat and muscle waste, reduced joint mobility correlated with increased levels of intra-articular and systemic pro-inflammatory cytokines. Despite a theoretically adequate diet, TNF-α and IL-1 β were found to increase resting energy expenditure (REE) and to alter body composition in RA patients (56). Cancer-associated systemic inflammation, indicated by the production of C reactive protein and fibrinogen, was associated with increased muscle catabolism, hypothalamic-driven anorexia, and increase in REE in cachectic patients (57). There are currently no available data to characterize the metabolic activity of AFP during extreme WAT pathological states. Description of an eventual independent AFP regulatory mechanism and/or its fluctuations in relation to systemic WAT has the potential to generate therapeutic targets for degenerative and inflammatory joint diseases such as OA and RA. Interestingly enough, both waste syndromes and diseases of excess WAT, regardless of their origin, are reported to benefit physical activity. Various regimens of exercise therapy are among the very few effective therapeutic interventions in cachexia, age-related lipodystrophy but as well in obesity, metabolic syndrome, and diabetes mellitus (54, 58). Recently, muscle mitochondrial activity and exercise-driven fibroblast growth factor 2 release were found to significantly reduce muscle mass and WAT loss in aging mice, linking muscle metabolism to both muscle and WAT maintenance during senescence (59). Moreover, diet and exercise are known to be efficient for the prevention and treatment of OA including in the non-weight-bearing joints (60).

Articular fat pad deposits remain quantitatively unmodified during extreme WAT states as a possible mechanism for preserving the joint homeostasis and hence enabling the body to remain mobile and to interact with the surroundings. In turn, preservation of mobility and the capability to engage in physical activities are mandatory for self-adjusting the equilibrium in multiple hierarchical systems inside and outside the body. AFP acting as a biomechanical sensor could adjust joint organ homeostasis by means of physical activity. Systemic WAT extremes are prevented and/or adjusted by mobility and so is the organism ability to interact within its ecosystem. Obviously, at the extremes, obesity and advanced wasting states overcome articular joint capability to self-adjustment by imposing severe external restrictions in mobility. Morbid obesity but as well lipodystrophy and cachexia mechanically restrict joint movements either by excessive body weight or by muscle wasting. Systemic or local inflammatory status and insulin resistance overcome the capability of AFP to maintain intra-articular homeostasis. Here, complex intervention that addresses both systemic conditions and intra-articular AFP impairment are needed.

Recently, intra-articular therapies using SVF and/or adipose tissue administration in OA joints are reporting favorable results in the management of knee OA (61, 62); however, their mechanism of action remains unknown. It is possible that such therapies act by recovering the HFP structural and functional balance that in turn contributes to restoring cellular turnover in several joint compartments and rehabilitate the metabolic and immune joint microenvironment. Intra-articular cell therapies could prove a disease modifying procedure to stop degradative processes during OA and RA.

We propose that AFP is an active component of the joint organ with multifunctional roles in maintenance of joint homeostasis. AFP rich network of sensitive nervous fibbers could act as a sensory organ possible involved in proprioception having role in acquiring information about joint axis, stability, and dynamics. Endocrine and paracrine secretion of adipokines and GFs AFP mature adipocytes could participate to joint organ turnover being involved in the maintenance of progenitor stem cell niche, cell renewal, and differentiation as well as local immune regulation. Local immune residents such as macrophage and Tregs are involved in balancing cellular growth and respond to pathological stimuli by controlling joint organ inflammatory status. While correlating with body-wide WAT status, AFP could possess genetic particularities as well as an independent mechanism of local control. Genetic and metabolic profiling of AFT could possibly result in description of molecular particularities that define distinct disease phenotypes. A metabolic-based classification of OA could result in predicting therapeutic response to existent preventive and therapeutic methods (63).

Further studies are needed to assess the biomechanical and molecular particularities of AFP in normal and diseased joints during normal and extreme WAT conditions. Cellular components as well as sensory fibers and ECM should be the subject of comparative investigation in both normal and pathological joint states as well as during normal and WAT pathological states. Using omics technologies at the single-cell level, complete AFP genetic and epigenetic profiling could be performed potentially deriving targets for future therapies. *In vivo* monitoring of AFP function (biomechanics, endocrine and paracrine release, and immune modulation) in animal models could elucidate its role within normal joint organ functioning and discriminate the contribution to the occurrence and progression of diseases. Bioinformatics analysis and computational modeling could identify currently unknown pathways involved in AFP functioning, eventually identifying AFP as an internal homeostatic system that connects joint biomechanics with structural maintenance mechanisms, correlated with systemic WAT but independently regulated. Cell therapies that aim to restore AFP structure and function could become the next step in delivering disease modifying therapies for disabling joint conditions such as OA and RA. Intra-articular therapies using adipose tissue derivatives might act by triggering AFP secretory and/or biomechanical role in regenerating joints structure and function.

## Conclusion

The presence and biomechanical importance AFP deposits are increasingly revealed due to the use of new and improved advanced dynamic imaging. Historically thought to possess a role in joint physiology by assumed production of joint lubricants, AFP metabolic role has been largely disregarded. WAT is increasingly recognized as an important endocrine organ with impact in body homeostasis. The similarity between WAT and articular fad pad regarding structure, cellularity, and composition invites to the reconsideration of its role in the maintenance of normal joint homeostasis. Methods that are designed to locally restore the functionality of the intra-articular adipose tissue could represent an effective modality to re balance joint homeostasis, improve joint function, and restore body mobility. This will derive important consequences for the treatment not solely for joint diseases but for extreme WAT misbalances—obesity, metabolic syndrome, age, and disease-associated wasting.

## Author Contributions

LL contributed to formulating and launching the hypothesis presented in this manuscript FZ-E contributed to gathering literature data and orienting manuscript writing.

## Conflict of Interest Statement

The authors declare that the research was conducted in the absence of any commercial or financial relationships that could be construed as a potential conflict of interest.
